# Anesthetic Management of Inguinal Hernia Surgery in an Adult Patient With Arthrogryposis Multiplex Congenita: A Case Report

**DOI:** 10.7759/cureus.80300

**Published:** 2025-03-09

**Authors:** Thalis Asimakopoulos, Panagiotis Prodromakis, Irene C Kouroukli

**Affiliations:** 1 Department of Anesthesiology and Pain Medicine, Hippocratio General Hospital of Athens, Athens, GRC

**Keywords:** airway, arthrogryposis multiplex congenita, dexmedetomidine, nerve blocks, perioperative management, regional anesthesia

## Abstract

Arthrogryposis multiplex congenita (AMC) poses major anesthetic challenges due to severe joint contractures, airway restrictions, and a debated risk of malignant hyperthermia. Despite these complexities, anesthetic reports in adult AMC patients remain extremely limited, leaving perioperative management largely unstandardized. We present the anesthetic approach for a 35-year-old male patient with AMC undergoing open inguinal hernia repair. Given the high risk of airway compromise and joint immobility, we opted for ultrasound-guided ilioinguinal and iliohypogastric nerve blocks with dexmedetomidine sedation, avoiding endotracheal intubation and volatile anesthetics. This case highlights regional anesthesia as a critical alternative to general anesthesia, providing valuable insights into safe, tailored anesthetic strategies for this rare and underreported population.

## Introduction

Arthrogryposis multiplex congenita (AMC) is an uncommon condition characterized by numerous nonprogressive contractures, affecting approximately one in 3,000 live births [1] with an equal gender ratio [2,3].

AMC is first diagnosed at birth and usually progresses until it reaches a state of significant disability. This condition manifests as nonprogressive limitations in joint mobility, prominently affecting the extremities and orofacial structures, often leading to microstomia and restricted temporomandibular joint motion [1,4]. There have also been cases of lethal arthrogryposis [5,6]. This syndrome is most commonly seen in children, with orthopedic surgeries being frequent, but sometimes adults may also present with it to undergo surgery.

The perioperative management of patients with AMC presents unique challenges due to these physical anomalies, including limited jaw movement and mouth opening, restricted lung development, intraoperative positioning issues, challenging venous access, and worries about an elevated risk of malignant hyperthermia [4]. Thus, traditional anesthesia techniques involving endotracheal intubation and volatile anesthetics carry heightened risks in this patient population [1,4]. Therefore, anesthetic strategies must be carefully tailored to address these challenges and minimize associated complications.

This report presents a case involving a 35-year-old male patient diagnosed with AMC who underwent inguinal hernia surgery. This case was previously presented at the 18th World Congress of Anesthesiologists held in Singapore from March 3, 2024, to March 7, 2024, as an e-poster.

## Case presentation

A 35-year-old male patient with a known history of AMC was scheduled for open left inguinal hernia surgery. The patient, weighing 23 kg, exhibited significant physical limitations, including severe contractures of the extremities and limited orofacial mobility, specifically microstomia and restricted temporomandibular joint motion, along with a Mallampati score III. These features raised concerns about airway management and venous access difficulties.

Preoperative evaluation highlighted the need for a careful anesthetic approach to avoid endotracheal intubation and the use of volatile anesthetics, considering the difficult airway and the potentially elevated risk of malignant hyperthermia, respectively [2].

**Figure 1:**
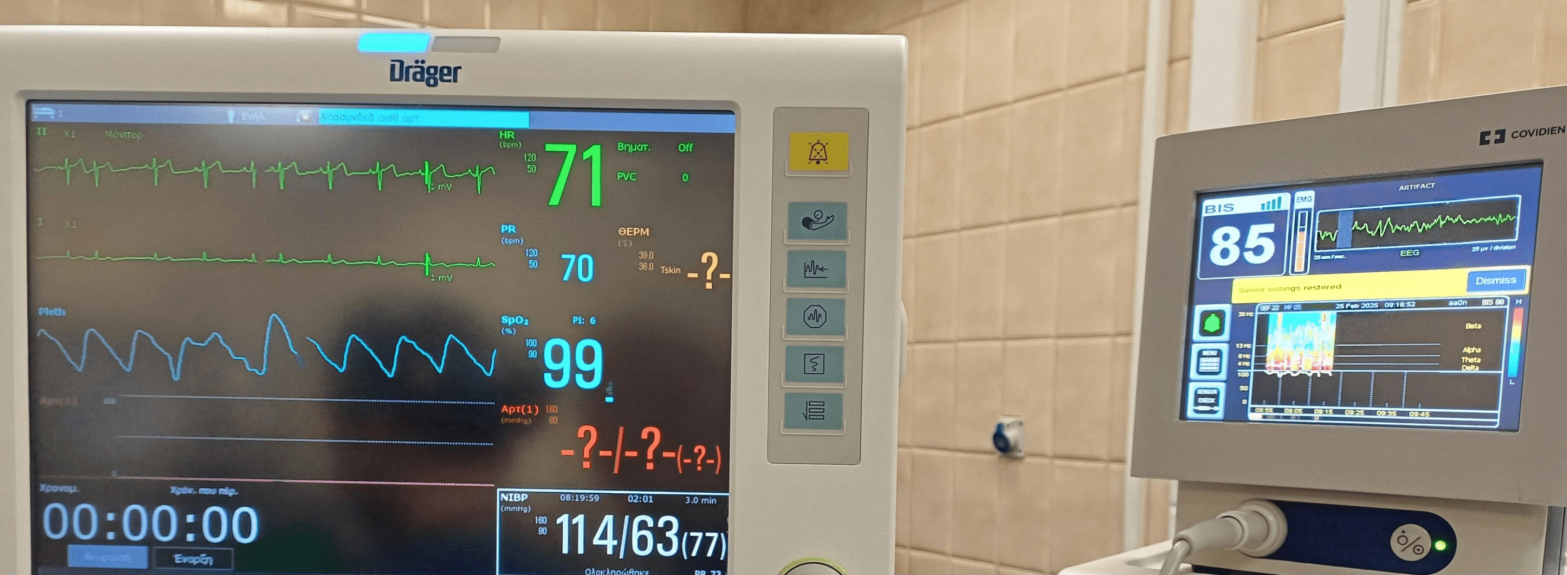
Comprehensive intraoperative monitoring setup

Basic monitoring, including electrocardiogram, noninvasive blood pressure, pulse oximetry, capnography, and bispectral index (BIS), was implemented to ensure comprehensive intraoperative monitoring (Figure 1). Perioperative monitoring of body temperature by skin temperature sensors was also applied. To ensure adequate oxygen supply, a Venturi mask at 30% FiO_2_ was used, with capnography monitoring to ensure that the patient was breathing spontaneously by watching the wave on the ventilator screen at all times.

**Figure 2:**
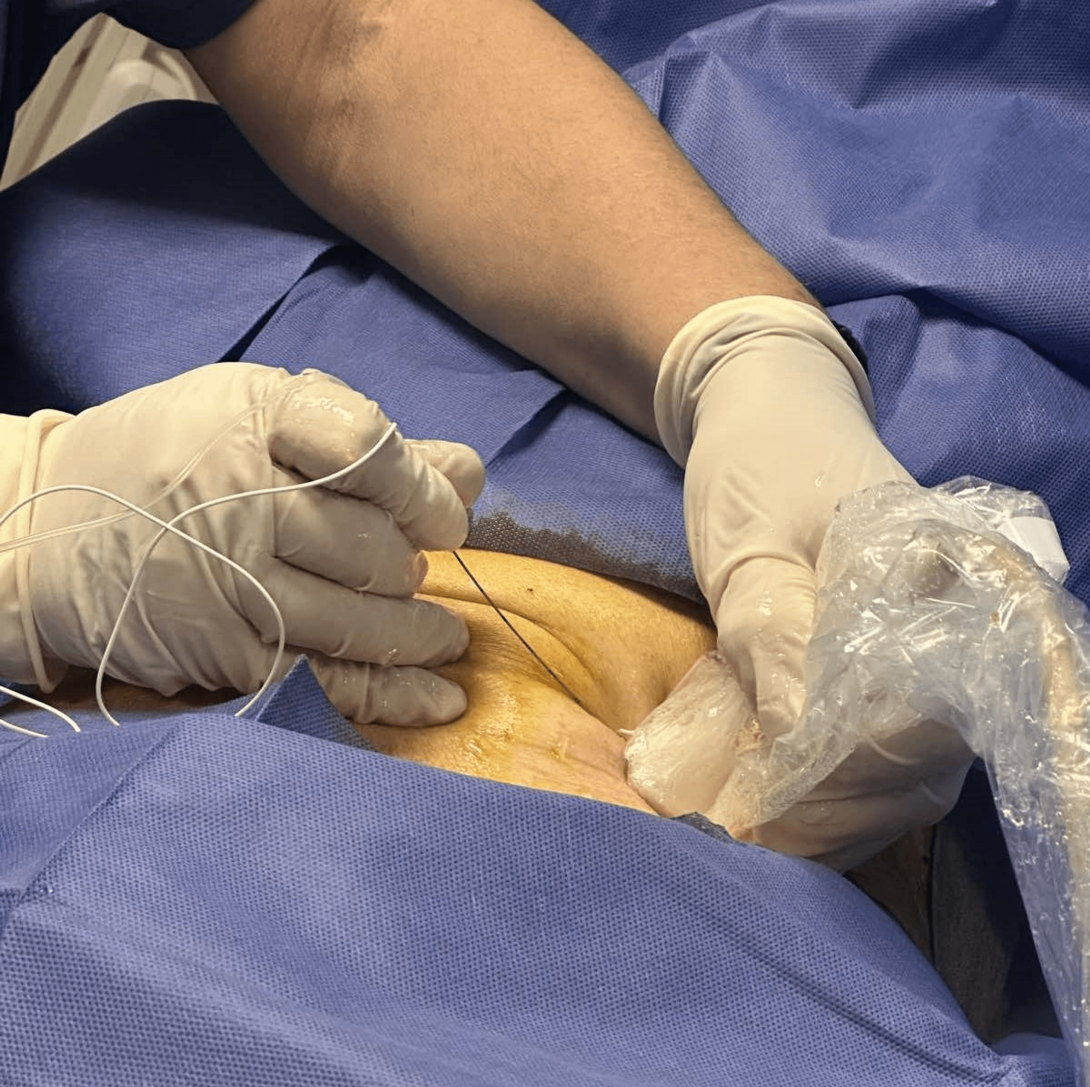
Ultrasound-guided ilioinguinal and iliohypogastric nerve block

Given the patient's anatomical limitations, ultrasound-guided blocks of the left ilioinguinal and iliohypogastric nerves were performed with the patient in a supine position using a 22G, 50-mm needle to administer 8 mL of 0.5% ropivacaine, ensuring effective intraoperative analgesia while adhering to safety guidelines for local anesthetic dosing. The ultrasound probe was placed over the anterior abdominal wall at the level of the inguinal ligament, and the injection was performed using an in-plane technique to confirm proper placement at the nerve target sites (Figure 2). To facilitate sedation and additional analgesia, a dexmedetomidine bolus of 1 mcg/kg was administered over 10 minutes, followed by a continuous infusion at 0.2-0.3 mcg/kg/hour. The patient's level of sedation was maintained at a Ramsay Sedation Scale score of 3-4 and BIS that ranged in values between 60 and 70, ensuring adequate sedation while allowing prompt response to commands.

Intraoperative analgesia was further supported by administering paracetamol (15 mg/kg) and dexketoprofen (50 mg IV over 20 minutes) 20 minutes before the conclusion of the surgery. The patient remained stable throughout the procedure and did not require additional analgesics in the postanesthesia care unit (PACU). He was discharged from the PACU 30 minutes after surgery without any adverse events. This approach provided effective pain management and smooth recovery, demonstrating the success of a tailored anesthetic strategy for this patient.

## Discussion

**Figure 3:**
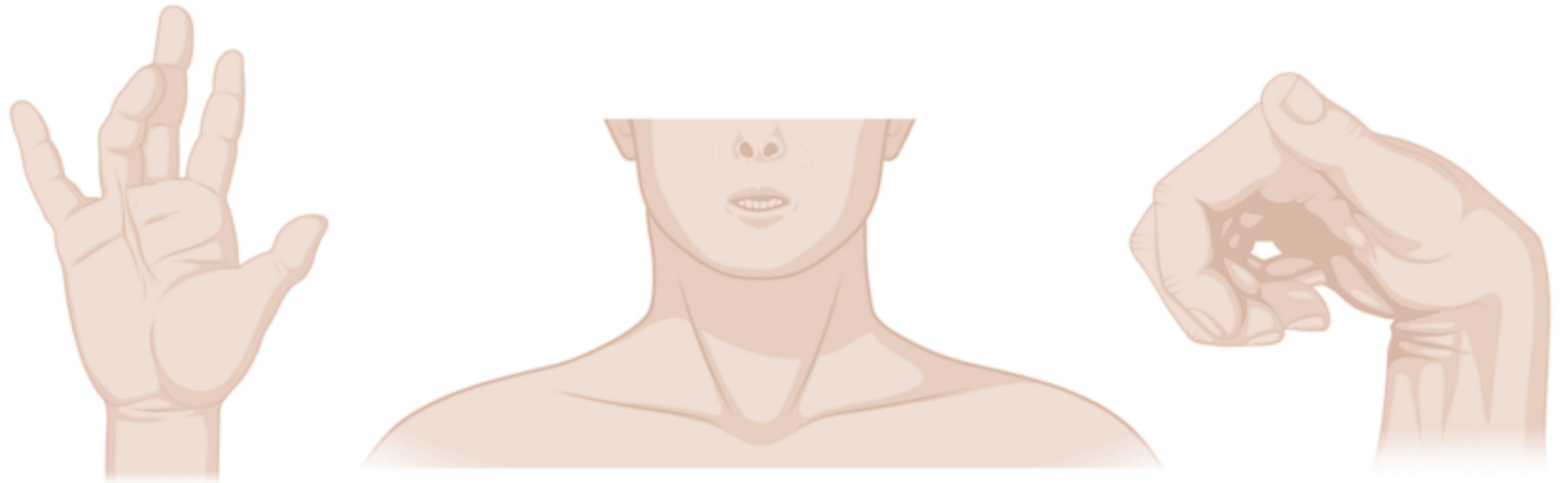
Visual depiction of characteristic anatomical features in AMC, including contractures of the extremities, restricted orofacial mobility (e.g., microstomia), and limited temporomandibular joint motion. These challenges necessitate tailored anesthetic approaches to ensure safe and effective perioperative management AMC: arthrogryposis multiplex congenita Image credit: This is an original image created by the author Thalis Asimakopoulos using BioRender (https://BioRender.com/f42s793)

An individualized anesthetic and analgesic approach was employed, including ultrasound-guided regional blocks and appropriate sedation, ensuring effective pain management throughout the procedure. The successful perioperative management of this patient with AMC highlights the importance of a tailored anesthetic and analgesic approach. There are different anesthetic considerations for AMC, primarily because each case has a unique etiology. Each pathophysiological abnormality must be considered from a different perspective due to its unique mechanism of disease (Figure 3) [7]. The literature is sparse regarding the most appropriate anesthetic management in this population, especially in adults. Table 1 outlines the different challenges faced by anesthesiologists.

**Table 1:** Anesthetic considerations in the patient with arthrogryposis multiplex congenita MH: malignant hyperthermia; AMC: arthrogryposis multiplex congenita; CSF: cerebrospinal fluid Source: Adapted from [7]

Anesthetic concerns	Abnormality
Airway	Micrognathia, cervical spine deformities, and high-arched palate
Induction agents	Increased sensitivity secondary to decreased muscle mass
Succinylcholine	Increased susceptibility to MH with myogenic causes of AMC
Regional block	Deformities of the vertebral column, abnormal spinal cords (decreased anterior horn cells), and abnormal CSF joint contractures: decreased accessibility to nerves

The decision to avoid general anesthesia and endotracheal intubation was based on the patient's significant jaw restriction and the associated risks of difficult airway management. In cases of difficulty with airway management, a laryngeal mask airway could be considered as a potential alternative to endotracheal intubation. However, given the patient's anatomical limitations and the risks associated with both general anesthesia and intubation, we prioritized regional anesthesia and sedation as the primary approach. Based on past literature, there is an associated risk for malignant hyperthermia, which does not seem to be corroborated with more current research. Our decision was consistent with findings from the literature, which emphasize the importance of individualized perioperative care for AMC patients [1,8].

Isaacson and Drum have also documented the complexities of airway management in AMC patients, particularly in those with severe jaw and limb contractures [4]. The use of regional anesthesia, as demonstrated in this case, provided effective pain control while minimizing the risks associated with general anesthesia, including intubation. The combination of regional blocks and dexmedetomidine sedation allowed effective analgesia and sedation, as seen in the study by Savenkov et al., who discussed the advantages of regional anesthesia in AMC patients [9].

Epidural anesthesia can also be a viable option for treating labor pain in patients with AMC; successful use of combined spinal and epidural anesthesia suggests this option [10]. Neuraxial anesthesia was the main choice in labor cases described in the literature. Quance [7] had to resort to general anesthesia after epidural failed to provide the necessary results, while Rozkowski et al. [11] and Spooner [12] managed to complete the procedure with the neuraxial technique alone. Early assessment of the identification of patients’ risks and comorbidities and thorough planning are recommended for safe anesthesia conduct [10].

This case underscores the need for individualized perioperative management strategies in AMC patients to address their unique anatomical and physiological challenges. In cases like this, interdisciplinary collaboration between surgeons, anesthesiologists, and the patient's personal physician is crucial for tailoring a safe and effective perioperative management plan. The successful outcome of this case adds to the growing body of evidence supporting the use of regional anesthesia and dexmedetomidine sedation in this patient population.

## Conclusions

Patients with AMC require highly individualized perioperative management strategies due to their distinct anatomical and physiological challenges. This case report provides valuable insights into the successful anesthetic management of a 35-year-old male patient with AMC undergoing open left inguinal hernia surgery. The use of regional anesthesia combined with dexmedetomidine sedation effectively addressed the patient's unique needs, ensuring a stable intraoperative course and a favorable postoperative outcome. These findings highlight the importance of customized anesthetic approaches in managing AMC patients and contribute to the broader understanding of perioperative care in this rare and underexplored condition.
